# Prevalence of allergen-specific IgE in southern China: a multicenter research

**DOI:** 10.18632/aging.203341

**Published:** 2021-07-22

**Authors:** Xue’an Wang, Long Zhou, Guixi Wei, Hui Zhang, Bin Yang

**Affiliations:** 1 Department of Laboratory Medicine, West China Hospital, Sichuan University, Chengdu 610041, China; 2 Department of Laboratory Medicine, Chongqing University Sanxia Hospital, Chongqing 404000, China; 3 Department of Laboratory Medicine, Liuzhou People's Hospital, Liuzhou 545000, China; 4 Department of Laboratory Medicine, Shenzhen Second People's Hospital, Shenzhen 518048, China

**Keywords:** food allergen, aeroallergen, prevalence, allergen-specific IgE, southern China

## Abstract

Identifying allergen distribution is meaningful and significant for effective diagnosis and treatment of allergic diseases. This study compared the allergen sensitivity in four southern China cities. We enrolled 55,432 participants (27,408 male, 28,024 female) between 2007 and 2019. The allergen-specific IgE levels were compared by the χ2 test. The five prevalent sensitivities were for mite mix (10,985, 19.82%), cockroach (4,860, 8.77%), crab (4,450, 8.03%), fish mix (3,874, 6.99%), and house dust (3,486, 6.29%). Almost all allergen sensitivities decreased with age, particularly from infant to middle aged participants (p < 0.05). An exception was Shenzhen, where food allergen positive rates remained constant in all age groups studied. The proportion of male sensitive to at least one food allergen (OR 1.130; 95% CI 1.088–1.174, p < 0.0025) or aeroallergen (OR, 1.117; 95% CI, 1.078–1.158, p < 0.0025) was higher than female in all four cities. Except for dog dander and tree mix, all aeroallergens differed significantly between seasons (p < 0.05). Liuzhou had the highest rates of food allergen- and aeroallergen-positive participants. The allergen-specific IgE distribution differed among the studied cities, with significant seasonal differences. Young age, male sex, and aeroallergens were risk factors for allergic disease.

## INTRODUCTION

The growing number of allergic diseases in recent decades worldwide has become a public health concern, for which the World Health Organization has prioritized prevention and treatment activities [1]. In 2016, 8.4% of the United States population had asthma compared to 4.3% globally [2]. A survey from 2010 covering eight provinces and cities in China found asthma prevalence of 1.24% among people older than 14 years [3]. The questionnaire-based self-reported prevalence of allergic rhinitis in 11 cities in China ranged between 8.5–24.1% in 2009 [4]. An epidemiological survey in Shanghai in 2012 showed that the prevalence of atopic dermatitis in children aged 3–6 years was 8.3% [5]. With changes in the environment and rapid economic development [6, 7], people in China pay more attention to allergies, but there is still no multicenter, large-scale study focusing on allergies in the Chinese population.

It is well known that allergic diseases are caused by the body immune-mediated overreaction to foreign proteins. The allergenicity of proteins is likely determined by additional factors, such as the amount and duration of exposure, environmental conditions, including microbial exposure, immune-modulating components of allergenic sources facilitating the Th2 immune response, and the intrinsic effects of proteins on the innate and adaptive immune systems [8]. Boulet et al. [9] used allergy skin prick tests for common airborne indoor and outdoor allergens in 3,371 consecutive patients and found that sensitization for indoor allergens was more prevalent than outdoor allergens in all age and diagnostic groups. The prevalence and degree of sensitization peaked in young adults, regardless of the allergen, and diminished with age. There is now compelling evidence that rising air temperatures and carbon dioxide concentrations result in increased pollen production, allergenicity, advancement, and lengthening of the pollen season in some plant species. Changes in extreme weather events, such as thunderstorms and tropical cyclones, could also impact allergic diseases. For example, flooding associated with tropical cyclones could lead to mold proliferation in damp homes [10]. Shah and Newcomb [11] found that asthma prevalence in boys was higher than in girls, while in adults, asthma was more prevalent in females than in males. Many factors, including genetic, environmental, immunological responses, and sex hormones, affect the sex disparity associated with the development and control of asthma and other allergic diseases. Fluctuations in hormones during puberty, menstruation, pregnancy, and menopause alter asthma symptoms and severity. Overall, the distribution of allergens differs by climate, geographic region, and lifestyle. Different age or sex groups have different sensitivities to allergens. However, understanding allergic diseases requires knowledge of the sources, distributions, and properties of individual allergens. An epidemiological survey of the distribution of common allergens in China is lacking.

Skin puncture tests, spot tests, and specific immunoglobulin E (IgE) tests are usually used to detect allergens. The specific IgE test is based on a combination of the antigen and the patient antigen-specific IgE. This test is advantageous as it does not lead to skin lesions and avoids other risks associated with allergic induction tests.

This multicenter study used antigen-specific IgE tests to investigate the distribution of common allergens in the southern China cities of Chengdu, Chongqing, Liuzhou, and Shenzhen.

## RESULTS

### The distinguishing feature of allergen distribution in Chengdu

**Figure 1:**
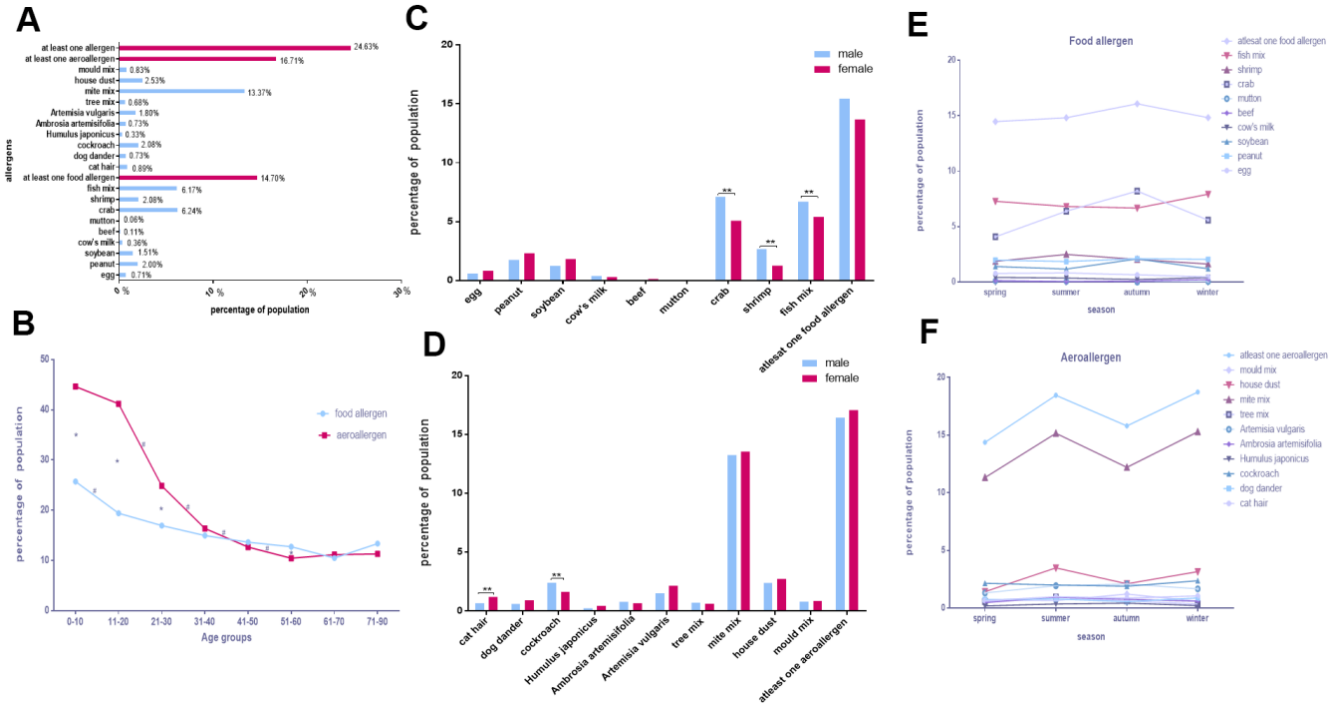
**The distinguishing feature of allergen distribution in Chengdu.** (**A**) The positive rates of various allergens. (**B**) Food allergen and aeroallergen positive rates in different age groups. (**C**, **D**) Comparison of allergen positive rates in sex. (**E**, **F**) Discrepancies of allergens with seasons.

Specific IgE tests were performed in 14,030 participants, 8,031 (57.24%) males and 5,999 (42.76%) females, aged 41.93 ± 14.49 years. Of these, 3,455 (24.63%) were sensitive to at least one allergen. The three most common allergens were mite mix (1,876, 13.37%), crab (876, 6.24%), and fish mix (865, 6.17%; Figure 1A).

As shown in Figure 1B, the incidence of allergies to food allergens and aeroallergens decreased with age, with significant differences in aeroallergens between the 11–20, 21–30, 31–40, 41–50, and 51–60 age groups. Only age groups 0–10 and 11–20 differed in food allergens. Furthermore, some age groups (0–10, 11–20, 21–30, and 51–60) differed in their sensitivity to food allergens and aeroallergens (*p* < 0.05). The positive rates of each allergen in all age groups in Chengdu are showed in Supplementary Figure 1A–1F. Significantly higher positive rates in males than females were observed for single allergen such as crab (odds ratio [OR], 1.429; 95% confidence interval [CI], 1.238–1.649; *p* < 0.0025), shrimp (OR, 2.116; 95% CI, 1.627–2.751; *p* < 0.0025), fish mix (OR, 1.252; 95% CI, 1.086–1.443; *p* < 0.0025), and cockroach (OR, 1.491; 95% CI, 1.166–1.905; *p* < 0.0025), while the situation was precisely the opposite in cat hair (OR, 0.547; 95% CI, 0.383–0.781; *p* < 0.0025; Figure 1C, 1D and Table 1).

**Table 1:** Difference of distribution of allergens between male and female participants in Chengdu.

**Allergens**	**Male**	**Female**	**P**	**OR**	**95%CI**
egg	0.61%	0.85%	0.095	0.716	0.483-1.061
peanut	1.76%	2.32%	0.019	0.753	0.595-0.955
soybean	1.27%	1.83%	0.007	0.689	0.525-0.904
cow's milk	0.39%	0.32%	0.496	1.220	0.688-2.161
beef	0.09%	0.15%	0.275	0.581	0.216-1.560
mutton	0.06%	0.05%	0.764	1.245	0.297-5.212
crab**	7.11%	5.08%	9.28E-07	1.429	1.238-1.649
shrimp**	2.68%	1.28%	1.06E-08	2.116	1.627-2.751
fish mix**	6.71%	5.43%	0.001859	1.252	1.086-1.443
at least one food allergen	15.45%	13.69%	0.003	1.153	1.048-1.268
cat hair**	0.66%	1.20%	0.000754	0.547	0.383-0.781
dog dander	0.60%	0.92%	0.028474	0.650	0.441-0.958
cockroach**	2.42%	1.63%	0.001327	1.491	1.166-1.905
*H*. japonicus	0.24%	0.45%	0.028636	0.525	0.291-0.944
*A*. artemisifolia	0.78%	0.67%	0.419191	1.178	0.791-1.753
*A*. vulgaris	1.53%	2.15%	0.006328	0.708	0.552-0.908
tree mix	0.72%	0.62%	0.451226	1.172	0.775-1.773
mite mix	13.24%	13.55%	0.586327	0.973	0.882-1.073
house dust	2.38%	2.73%	0.184661	0.867	0.702-1.071
mold mix	0.81%	0.87%	0.711244	0.933	0.647-1.346
at least one aeroallergen	16.44%	17.07%	0.319891	0.956	0.874-1.045

**p < 0.0025 was considered statically significant.

OR: odds ratio.

The three allergens with the most positive rates in every season in Chengdu were mite mix, fish mix, and crab. Furthermore, the positive rates of soybean, crab, cat hair, tree mix, mite mix, house dust and mold mix vary with the season. (*p* < 0.05; Figure 1E, 1F and Tables 2, 3).

**Table 2:** Discrepancies of food allergens in these cities between seasons.

	**Egg**	**Peanut**	**Soybean**	**Cow's milk**	**Beef**	**Mutton**	**Crab**	**Shrimp**	**Fish mix**	**At least one food allergen**
p (Chengdu)	0.371	0.85049	0.002819	0.512493	0.17479	0.153624	3.6E-12	0.08327	0.256785	0.207483
spring	0.78%	1.99%	1.41%	0.43%	0.14%	0.12%	4.09%	1.87%	7.29%	14.47%
summer	0.83%	1.85%	1.18%	0.37%	0.05%	0.07%	6.40%	2.50%	6.82%	14.82%
autumn	0.66%	2.12%	2.10%	0.24%	0.10%	0.00%	8.21%	2.05%	6.68%	16.08%
winter	0.47%	2.05%	1.21%	0.42%	0.23%	0.05%	5.59%	1.63%	7.93%	14.83%
p (Chongqing)	0.441374	0.4532	0.386068	0.674922	0.652987	0.58443	0.26103	0.36971	0.308746	0.266285
spring	1.10%	3.63%	4.95%	0.58%	0.50%	0.10%	5.66%	2.45%	6.96%	22.32%
summer	1.03%	3.76%	4.18%	0.57%	0.55%	0.05%	5.94%	2.50%	6.17%	20.56%
autumn	1.22%	3.09%	4.58%	0.39%	0.61%	0.03%	6.77%	3.06%	5.93%	21.14%
winter	1.44%	3.43%	4.41%	0.53%	0.39%	0.11%	6.16%	2.77%	6.41%	21.40%
p (Liuzhou)	0.174388	0.07486	3.91E-18	0.001172	3.34E-12	0.072527	0.00001	0.14546	1.12E-07	3.22E-07
spring	9.81%	3.19%	7.97%	11.34%	6.11%	2.29%	11.26%	3.55%	7.80%	34.71%
summer	10.28%	3.41%	7.61%	9.01%	4.56%	1.60%	14.07%	3.79%	8.35%	35.28%
autumn	9.96%	3.92%	11.33%	10.14%	6.71%	1.92%	12.40%	3.54%	9.96%	39.01%
winter	8.81%	4.17%	12.53%	10.24%	8.52%	2.07%	11.01%	2.84%	11.27%	39.65%
p (Shenzhen)	0.000505	0.03433	0.461637	0.000066	0.089658	0.300757	0.29713	0.31027	0.002999	0.032486
spring	5.56%	1.64%	4.37%	5.19%	2.41%	1.00%	9.66%	3.19%	7.29%	24.83%
summer	6.22%	2.51%	4.06%	4.75%	2.72%	1.25%	10.54%	3.07%	7.04%	25.23%
autumn	5.92%	1.99%	4.94%	5.49%	3.31%	1.36%	11.05%	3.11%	5.14%	25.26%
winter	3.76%	1.41%	4.24%	2.95%	2.22%	0.85%	9.69%	2.38%	5.57%	22.17%
p (Four cities)	0.001762	0.75168	4.16E-08	0.000002	5.23E-07	0.074117	3.09E-08	0.06498	0.051316	0.393311
spring	4.70%	2.80%	5.08%	4.95%	2.64%	1.00%	7.82%	2.80%	7.38%	25.13%
summer	4.82%	2.97%	4.54%	3.94%	2.07%	0.74%	9.44%	3.01%	7.19%	24.67%
autumn	4.24%	2.79%	5.64%	3.87%	2.56%	0.77%	9.52%	2.88%	7.06%	25.02%
winter	3.94%	2.89%	6.10%	3.92%	3.17%	0.84%	8.29%	2.47%	7.94%	25.59%

*p* < 0.05 was considered statically significant.

**Table 3:** Discrepancies of aeroallergens in these cities between seasons.

	**Cat hair**	**Dog dander**	**Cockroach**	***H.* japonicus**	***A*. artemisifolia**	***A.* vulgaris**	**Tree mix**	**Mite mix**	**House dust**	**Mold mix**	**At least one aeroallergen**
p (Chengdu)	1.7E-07	0.433172	0.618812	0.265157	0.10184	0.066911	0.03867	7.2E-08	1.417E-08	0.014231	6.248E-07
spring	0.66%	0.75%	2.16%	0.20%	0.49%	1.33%	0.58%	11.33%	1.44%	0.72%	14.38%
summer	1.00%	0.81%	2.02%	0.37%	0.95%	1.95%	0.95%	15.16%	3.50%	0.67%	18.46%
autumn	0.88%	0.56%	1.90%	0.44%	0.78%	2.10%	0.63%	12.21%	2.12%	1.22%	15.81%
winter	1.07%	0.89%	2.38%	0.23%	0.61%	1.68%	0.37%	15.29%	3.17%	0.61%	18.74%
p (Chongqing)	0.09286	0.297607	0.277379	0.623658	0.930804	0.610658	0.27042	0.60867	0.393543	0.844836	0.153423
spring	3.15%	3.55%	6.06%	0.35%	1.55%	1.83%	1.48%	13.61%	9.01%	1.35%	31.71%
summer	2.98%	3.88%	7.11%	0.50%	1.70%	2.20%	1.93%	14.62%	8.51%	1.49%	33.75%
autumn	2.48%	3.74%	6.48%	0.55%	1.71%	1.97%	1.42%	13.92%	8.12%	1.51%	32.13%
winter	2.28%	3.05%	6.58%	0.46%	1.58%	2.17%	1.72%	14.15%	7.95%	1.61%	31.70%
p (Liuzhou)	0.00042	0.000002	1.48E-28	0.000181	0.000025	1.62E-07	0.23479	0.08925	1.62E-09	3.60E-10	0.011715
spring	4.13%	2.15%	15.77%	1.55%	3.68%	5.41%	3.64%	37.08%	6.35%	2.37%	49.62%
summer	3.03%	0.97%	14.47%	1.27%	2.80%	3.68%	3.70%	38.59%	9.73%	1.87%	49.73%
autumn	2.73%	1.45%	21.46%	2.29%	4.18%	3.95%	4.33%	38.66%	8.89%	3.57%	52.60%
winter	4.00%	1.10%	22.34%	2.29%	4.71%	5.97%	4.26%	36.33%	7.68%	4.04%	51.82%
p (Shenzhen)	0.03275	0.553275	0.021847	0.023533	0.354019	0.206748	0.05855	1.6E-05	0.001344	0.010409	0.000003
spring	3.78%	1.91%	8.56%	1.14%	3.23%	4.10%	3.83%	31.71%	5.56%	1.32%	40.68%
summer	5.53%	1.90%	10.89%	1.08%	3.20%	3.97%	4.62%	36.98%	6.87%	2.68%	46.05%
autumn	4.36%	2.37%	10.67%	1.44%	4.01%	4.44%	4.20%	37.84%	8.45%	2.45%	48.15%
winter	4.28%	1.90%	9.29%	0.57%	3.72%	5.13%	3.15%	33.80%	6.74%	2.14%	44.10%
p (Four cities)	0.01103	0.073662	1.05E-09	0.015348	0.001252	0.00004	0.45562	0.0001	4.90E-07	0.000002	0.000013
spring	3.00%	2.17%	8.87%	0.85%	2.29%	3.28%	2.36%	23.88%	5.80%	1.55%	35.16%
summer	2.84%	1.82%	8.79%	0.81%	2.08%	2.88%	2.65%	25.95%	7.39%	1.57%	36.86%
autumn	2.41%	1.89%	9.77%	1.14%	2.51%	3.00%	2.47%	24.48%	6.52%	2.14%	35.51%
winter	3.01%	1.77%	10.98%	0.97%	2.80%	3.88%	2.53%	25.48%	6.62%	2.24%	37.87%

*p* < 0.05 was considered statically significant.

### The property of allergen distribution in Chongqing

**Figure 2:**
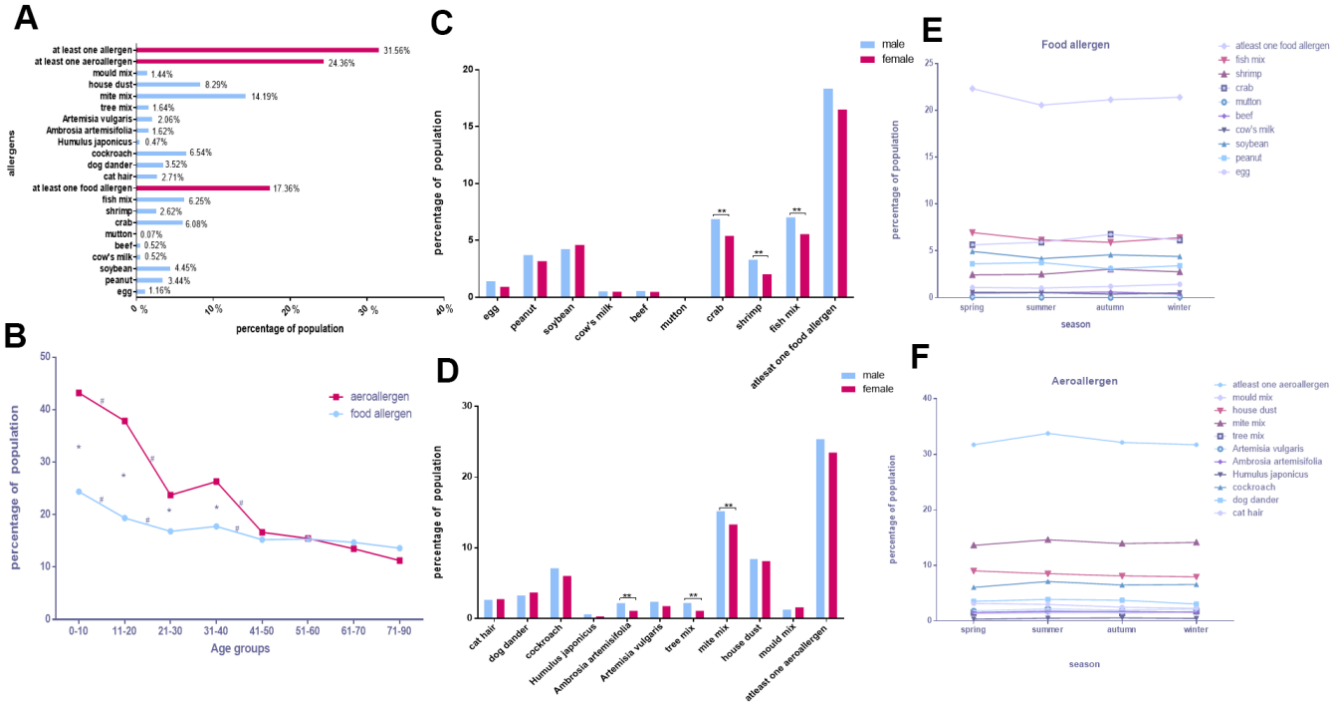
**The property of allergen distribution in Chongqing.** (**A**) The positive rates of various allergens. (**B**) Food allergen and aeroallergen positive rates in different age groups. (**C**, **D**) Comparison of allergen positive rates in sex. (**E**, **F**) Discrepancies of allergens with seasons.

Of the 14,612 participants in Chongqing (46.41% male, 53.59% female), 31.56% were sensitive to at least one allergen. The single allergens with the top five sensitivities were mite mix (14.19%), house dust (8.29%), cockroach (6.54%), fish mix (6.25%), and crab (6.08%; Figure 2A).

The proportion of participants positive for aeroallergens was higher than for food allergens in the 0–10, 11–20, 21–30, and 31–40 age groups. As shown in Figure 2B, the prevalence of aeroallergens and food allergens decreased with age, with a decrease in both in the 0–10, 11–20, and 21–30 age groups (*p* < 0.05). However, the positive rates for single allergens such as mite mix and crab increased from 0–10 to 11–20 (*p* < 0.05) and then decreased with age (Supplementary Figure 2C, 2F). And the other allergens situation in all age groups in Chongqing is showed in Supplementary Figure 2A, 2B, 2D, 2E.

Some single allergens showed remarkably higher levels in males than in females. These included crab (OR, 1.292; 95% CI, 1.128–1.480), fish mix (OR, 1.283; 95% CI, 1.122–1.467), shrimp (OR, 1.648; 95% CI, 1.341–2.025), mite mix (OR, 1.165; 95% CI, 1.062–1.279), tree mix (OR, 2.041; 95% CI, 1.565–2.661), and *Ambrosia artemisifolia* (OR, 2.037; 95% CI, 1.559–2.661; *p* < 0.0025 for all; Figure 2C, 2D and Table 4).

**Table 4:** Difference of distribution of allergens between male and female participants in Chongqing.

**Allergens**	**Male**	**Female**	**P**	**OR**	**95%CI**
egg	1.42%	0.93%	0.006423	1.526	1.124-2.072
peanut	3.72%	3.19%	0.083	1.170	0.979-1.398
soybean	4.26%	4.61%	0.308	0.921	0.786-1.079
cow's milk	0.53%	0.51%	0.867	1.039	0.662-1.633
beef	0.56%	0.49%	0.529	1.156	0.736-1.814
mutton	0.06%	0.08%	0.76	0.770	0.217-2.729
crab**	6.87%	5.40%	0.00021	1.292	1.1281.480
shrimp**	3.30%	2.03%	0.000002	1.648	1.341-2.025
fish mix**	7.03%	5.57%	0.000261	1.283	1.122-1.467
at least one food allergen	18.36%	16.50%	0.003	1.138	1.045-1.240
cat hair	2.67%	2.75%	0.776056	0.971	0.795-1.187
dog dander	3.29%	3.73%	0.149733	0.878	0.735-1.048
cockroach	7.12%	6.03%	0.007584	1.196	1.048-1.363
*H*. japonicus	0.63%	0.32%	0.005295	1.992	1.216-3.265
*A*. artemisifolia**	2.21%	1.10%	1.01E-07	2.037	1.559-2.661
*A*. vulgaris	2.39%	1.78%	0.009189	1.354	1.077-1.703
tree mix**	2.24%	1.11%	7.77E-08	2.041	1.565-2.661
mite mix **	15.19%	13.32%	0.001243	1.165	1.062-1.279
house dust	8.46%	8.14%	4.71E-01	1.044	0.928-1.175
mold mix	1.27%	1.60%	0.097	0.792	0.600-1.044
at least one aeroallergen	25.39%	23.47%	0.006995	1.110	1.029-1.197

**p < 0.0025 was considered statically significant.

OR: odds ratio.

It is worth mentioning that the allergen included in our study did not differ between seasons in Chongqing (*p* > 0.05; Figure 2E, 2F and Tables 2, 3).

### General characteristics of allergen distribution Liuzhou

**Figure 3:**
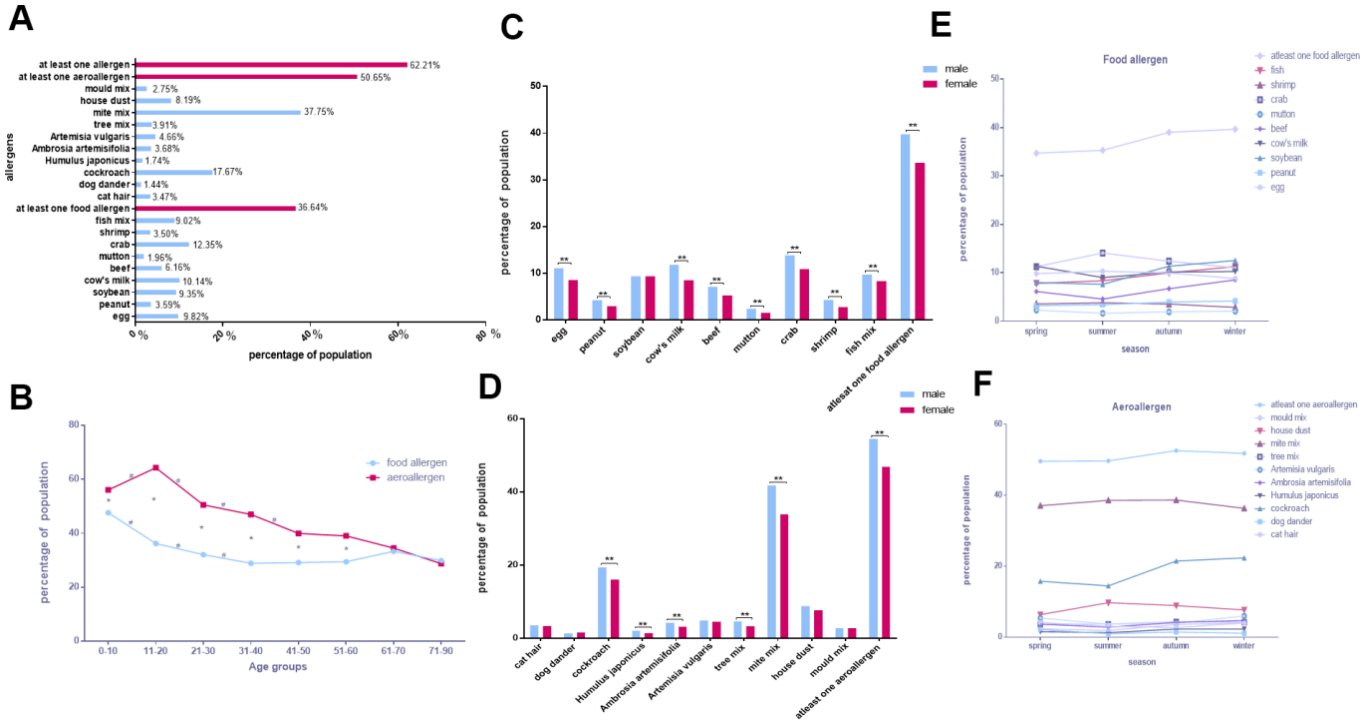
**General characteristics of allergen distribution Liuzhou.** (**A**) The positive rates of various allergens. (**B**) Food allergen and aeroallergen positive rates in different age groups. (**C**, **D**) Comparison of allergen positive rates in sex. (**E**, **F**) Discrepancies of allergens with seasons.

Surprisingly, we found that over half (62.21%) of the 17,217 participants in Liuzhou (48.98% male, 51.02% female) tested positive for allergen-specific antibodies. The percentages of allergies were as follows: mite mix, 37.75%; cockroaches, 17.67%; crab, 12.35%; cow’s milk, 10.14%; egg, 9.82%; soybean, 9.35%; fish mix, 9.02%; house dust, 8.19% (Figure 3A).

As shown in Figure 3B, the proportions of participants positive for aeroallergens and food allergen tended to decrease with age; however, an increasing trend was found from 0–10 to 11–20 for aeroallergens (*p* < 0.05). The aeroallergen positivity rate was higher than for food allergens in the first six age groups (*p* < 0.05). The positive rates of each allergen in all age groups in Liuzhou are showed in Supplementary Figure 3A–3F.

Some single allergens, including soybean, cat hair, dog dander, house dust, mold mix, and *Artemisia vulgaris*, had similar positivity rates in both sexes, while others always tested higher in males (*p* < 0.0025; Figure 3C, 3D and Table 5).

**Table 5:** Difference of distribution of allergens between male and female participants in Liuzhou.

**Allergens**	**Male**	**Female**	**P**	**OR**	**95%CI**
Egg**	11.12%	8.56%	1.62E-08	1.337	1.208-1.479
peanut**	4.23%	2.97%	0.000009	1.444	1.227-1.698
soybean	9.34%	9.35%	0.995871	1.000	0.902-1.108
cow's milk**	11.81%	8.54%	1.15E-12	1.435	1.298-1.585
beef**	7.09%	5.27%	6.87E-07	1.372	1.210-1.555
mutton**	2.40%	1.54%	0.000048	1.572	1.262-1.959
crab**	13.89%	10.88%	0.000	1.320	1.205-1.446
shrimp**	4.29%	2.74%	3.26E-08	1.590	1.347-1.877
fish mix**	9.74%	8.33%	0.001324	1.186	1.069-1.317
at least one food allergen**	39.75%	33.65%	1.04E-16	1.301	1.222-1.384
cat hair	3.58%	3.37%	0.449	1.065	0.905-1.254
dog dander	1.32%	1.56%	0.18	0.842	0.654-1.083
cockroach**	19.36%	16.04%	1.09E-08	1.257	1.162-1.360
*H*. japonicus**	2.06%	1.43%	0.001618	1.448	1.149-1.824
*A*. artemisifolia**	4.21%	3.18%	0.000319	1.340	1.142-1.572
*A*. vulgaris	4.84%	4.50%	0.288	1.080	0.937-1.244
tree mix**	4.59%	3.26%	0.000006	1.429	1.223-1.670
mite mix**	41.78%	33.88%	1.20E-26	1.400	1.316-1.490
house dust	8.78%	7.63%	0.00605	1.165	1.045-1.299
mold mix	2.72%	2.78%	0.803	0.977	0.814-1.173
at least one aeroallergen**	54.54%	46.91%	1.55E-23	1.357	1.278-1.441

**p < 0.0025 was considered statically significant.

OR: odds ratio.

The positive rates of soybean, cow's milk, beef, crab and fish mix vary with the season. (Figure 3E, Table 2). Except for tree mix and mite mix, all aeroallergens differed significantly between seasons (Figure 3F and Table 3). The positive rates for aeroallergens such as cockroach, *Humulus japonicus*, *A. artemisifolia*, *A. vulgaris*, and mold mix were the highest in winter, cat hair and dog dander were the highest in spring, and house dust was the highest in the summer (*p* < 0.05).

### The feature of the distribution of allergens in Shenzhen

**Figure 4:**
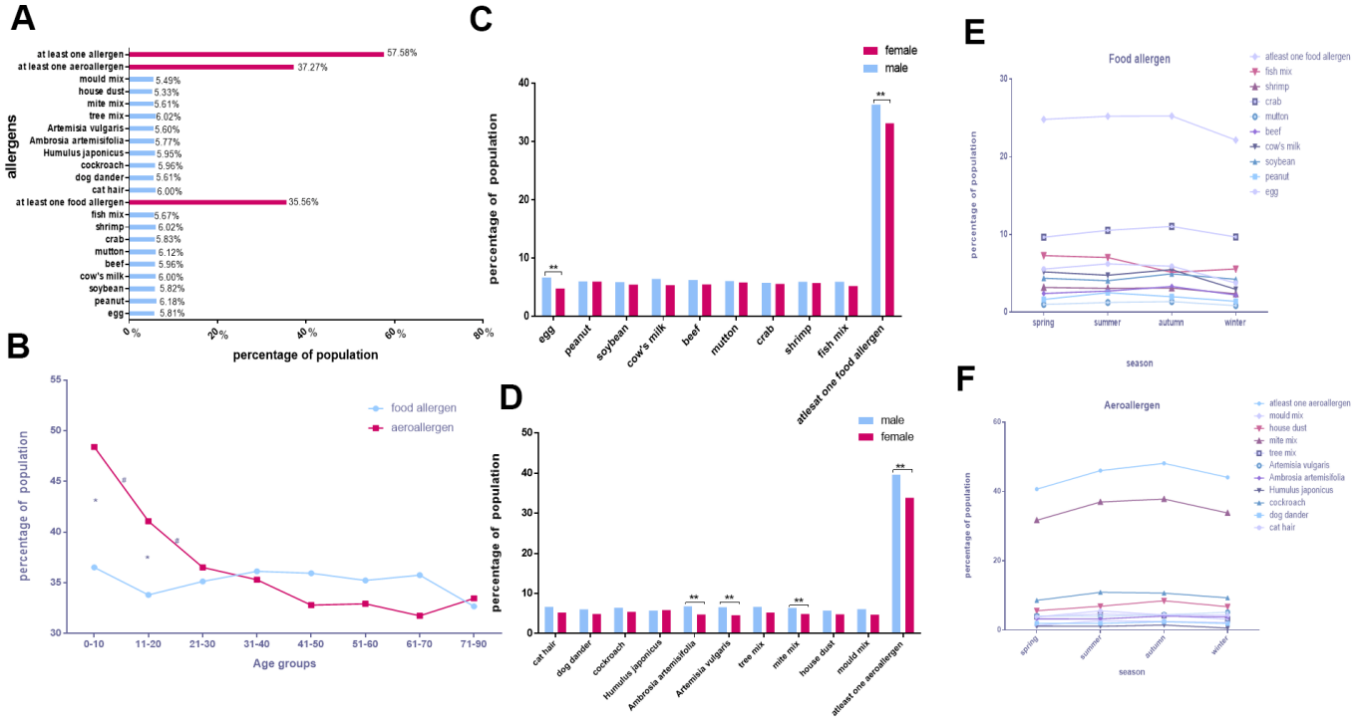
**The feature of the distribution of allergens in Shenzhen.** (**A**) The positive rates of various allergens. (**B**) Food allergen and aeroallergen positive rates in different age groups. (**C**, **D**) Comparison of allergen positive rates in sex. (**E**, **F**) Discrepancies of allergens with seasons.

We enrolled 9,573 participants in Shenzhen. Around one-third of them had IgE specific for food allergens (35.56%) or aeroallergens (37.27%). Intriguingly, almost all types of single allergens showed a similar positive rate (5.33–6.18%; Figure 4A).

All age groups showed a similar proportion of sensitivity to food allergens (*p* > 0.05). Only the 0–10, 11–20, and 21–30 age groups showed a downward trend in aeroallergens (*p* < 0.05). Additionally, the positive percentage of aeroallergens was higher than food allergens in the 0–10 and 11–20 age groups (*p* < 0.05; Figure 4B). The positive rates of each allergen in all age groups in Shenzhen are showed in Supplementary Figure 4A–4F.

The prevalence of sensitization to egg (OR, 1.407; 95% CI, 1.182–1.674; *p* < 0.0025), mite mix (OR, 1.326; 95% CI, 1.113–1.580; *p* < 0.0025), *A. vulgaris* (OR, 1.461; 95% CI, 1.225–1.742; *p* < 0.0025), and *A. artemisifolia* (OR, 1.451; 95% CI, 1.220–1.726; *p* < 0.0025) was remarkably higher in males than females (Figure 4C, 4D and Table 6).

**Table 6:** Difference of distribution of allergens between male and female participants in Shenzhen.

**Allergens**	**Male**	**Female**	**P**	**OR**	**95%CI**
egg**	6.68%	4.84%	0.000112	1.407	1.182-1.674
peanut	6.05%	5.97%	0.86	1.015	0.857-1.203
soybean	5.89%	5.47%	0.38	1.081	0.908-1.287
cow's milk	6.44%	5.38%	0.03	1.211	1.020-1.437
beef	6.30%	5.49%	0.1	1.157	0.975-1.373
mutton	6.08%	5.84%	0.62	1.044	0.880-1.238
crab	5.79%	5.58%	0.67	1.04	0.873-1.238
shrimp	5.96%	5.73%	0.64	1.043	0.878-1.238
fish mix	5.96%	5.23%	0.12	1.148	0.963-1.368
at least one food allergen**	36.38%	33.15%	0.001	1.153	1.059-1.255
cat hair	6.66%	5.30%	0.005	1.273	1.074-1.509
dog dander	6.05%	4.95%	0.02	1.237	1.036-1.476
cockroach	6.46%	5.41%	0.03	1.207	1.018-1.432
*H*. japonicus	5.74%	5.86%	0.81	0.979	0.823-1.164
*A*. artemisifolia**	6.80%	4.79%	0.000024	1.451	1.220-1.726
*A*. vulgaris**	6.61%	4.62%	2.30122E-05	1.461	1.225-1.742
tree mix	6.66%	5.25%	0.004	1.287	1.085-1.527
mite mix**	6.39%	4.90%	0.002	1.326	1.113-1.580
house dust	5.74%	4.82%	0.045	1.202	1.004-1.440
mold mix	6.10%	4.77%	0.004	1.298	1.086-1.551
at least one aeroallergen**	39.64%	33.89%	6.7497E-09	1.281	1.178-1.393

**p < 0.0025 was considered statically significant.

OR: odds ratio.

The positive rates of egg, peanut, cow's milk, fish mix and cat hair, cockroach, mold mix, *H. japonicus*, mite mix, house dust vary with the season. (P<0.05; Figure 4E, 4F and Tables 2, 3).

### Overall allergen distribution characteristics in the four cities

**Figure 5:**
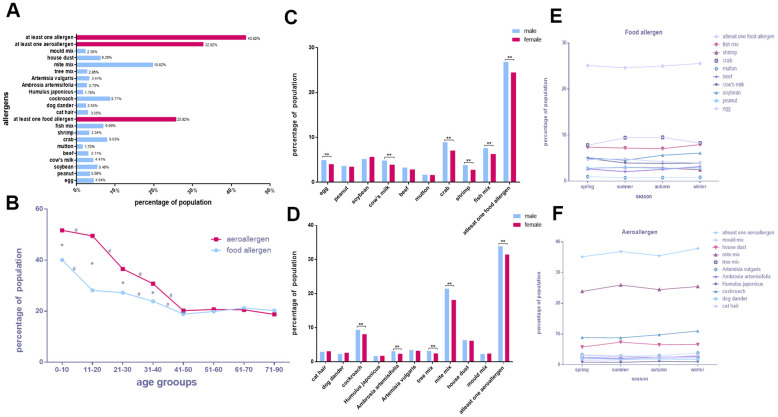
**Overall allergen distribution characteristics in the four cities.** (**A**) The positive rates of various allergens. (**B**) Food allergen and aeroallergen positive rates in different age groups. (**C**, **D**) Comparison of allergen positive rates in sex. (**E**, **F**) Discrepancies of allergens with seasons.

Of the 55,432 participants, 24,289 (48.32%) were sensitive to at least one of the tested allergens. The most prevalent allergens were mite mix (10,985, 19.82%), followed by cockroach (4,860, 8.77%), crab (4,450, 8.03%), fish mix (3,874, 6.99%), house dust (3,486, 6.29%), and soybean (3,028, 5.46%; Figure 5A).

General decreasing trends with age were noted in the sensitivity to food allergens and aeroallergens. Significant differences were noted between the 0–10, 11–20, 21–30, 31–40, and 41–50 age groups in aeroallergens (*p* < 0.05; Figure 5B). Furthermore, we found that the positive rate of sensitivity to aeroallergens was higher than for food allergens in the low and middle age groups (0–10, 11–20, 21–30, 31–40, and 41–50). The positive rates of each allergen in all age groups in all the participants are showed in Supplementary Figure 5A–5F.

The distributions of five food allergens [egg (OR, 1.235; 95% CI, 1.139–1.338), cow’s milk (OR, 1.249; 95% CI, 1.151–1.356), crab (OR, 1.286; 95% CI, 1.210–1.368), shrimp (OR, 1.377; 95% CI, 1.254–1.513), and fish mix (OR, 1.216; 95% CI, 1.139–1.299); *p* < 0.0025 for all] and four aeroallergens [cockroach (OR, 1.177; 95% CI, 1.110–1.249), *A. artemisifolia* (OR, 1.320; 95% CI, 1.191–1.464)*,* tree mix (OR, 1.297; 95% CI, 1.172–1.435), and mite mix (OR, 1.229; 95% CI, 1.179–1.282); *p* < 0.0025 for all] were significantly higher in males than females. Other allergens had similar rates in both sexes (*p* > 0.0025 for all; (Figure 5C, 5D and Table 7).

**Table 7:** Difference of distribution of allergens between male and female participants overall these four cities.

**Allergens**	**Male**	**Female**	**P**	**OR**	**95%CI**
Egg**	4.97%	4.06%	2.83E-07	1.235	1.139-1.338
peanut	3.66%	3.47%	0.243	1.055	0.964-1.154
soybean	5.20%	5.67%	0.014	0.912	0.848-0.982
cow's milk**	4.86%	3.93%	8.65E-08	1.249	1.151-1.356
beef	3.30%	2.88%	0.004054	1.152	1.046-1.268
mutton	1.69%	1.64%	0.636	1.032	0.906-1.175
Crab**	8.94%	7.09%	1.04E-15	1.286	1.210-1.368
Shrimp**	3.83%	2.81%	2.03E-11	1.377	1.254-1.513
fish mix**	7.61%	6.34%	4.74E-09	1.216	1.139-1.299
at least one food allergen**	26.83%	24.49%	3.0575E-10	1.130	1.088-1.174
cat hair	2.97%	3.10%	0.343	0.954	0.866-1.051
dog dander	2.31%	2.68%	0.005	0.859	0.772-0.956
Cockroach**	9.41%	8.11%	5.75E-08	1.177	1.110-1.249
*H*. japonicus	1.73%	1.77%	0.766	0.981	0.864-1.114
*A*. artemisifolia**	3.11%	2.37%	1.09E-07	1.320	1.191-1.464
*A*. vulgaris	3.53%	3.26%	0.075	1.087	0.992-1.192
tree mix**	3.19%	2.48%	4.2185E-07	1.297	1.172-1.435
mite mix**	21.46%	18.19%	4.08E-22	1.229	1.179-1.282
house dust	6.36%	6.18%	0.374	1.032	0.963-1.105
mold mix	2.31%	2.42%	0.396	0.954	0.855-1.064
at least one aeroallergen**	33.90%	31.46%	9.45E-10	1.117	1.078-1.158

**p < 0.0025 was considered statically significant.

OR: odds ratio.

Regardless of the season, the top five allergens among all study participants were mite mix, cockroach, crab, fish mix, and house dust. The single allergens cow's milk kept the highest positive rate in spring. Egg, mite mix and house dust maintained the highest positive rates in the summer, while soybean, beef, cat hair, *A. artemisifolia, A. vulgaris*, cockroach and mold mix showed the highest positive rates in the winter. Moreover, sensitivity to crabs and *H. japonicus* surged in the autumn (*p* < 0.05; Figure 5E, 5F and Tables 2, 3).

### Comparison of allergen positive rates between the four cities

**Figure 6:**
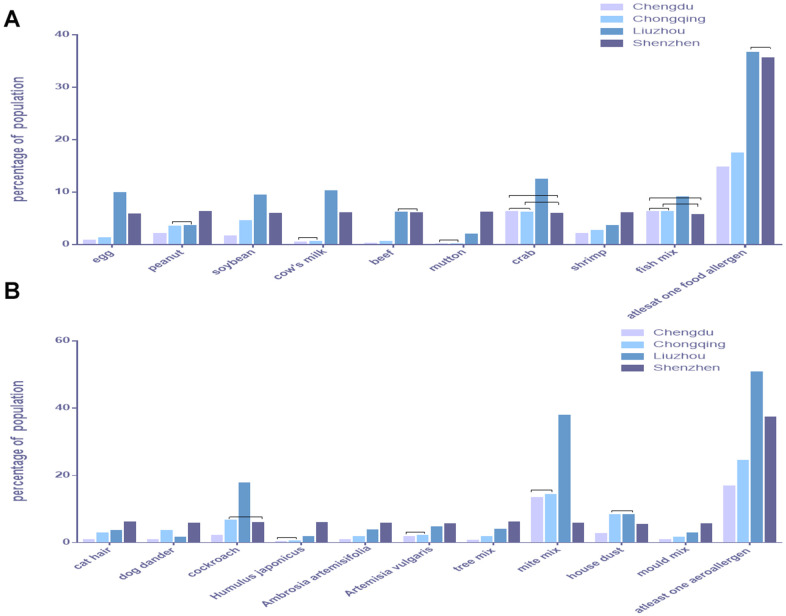
**Comparison of allergen positive rates between the four cities.** (**A**) Food allergens. (**B**) Aeroallergens.

Our extensive data confirmed that the proportion of allergies to all allergen types differed significantly between the four cities. The positive rate for egg and soybean allergens were highest in Liuzhou, followed by Shenzhen, Chongqing, and Chengdu (*p* < 0.0083), based on χ^2^ tests with Bonferroni correction for multiple comparisons. The positive rates of crab and fish mix were the highest in Liuzhou and similar among the other three cities (*p* > 0.0083; Figure 6A and Table 8).

**Table 8:** Differences of distribution of allergens between these four southern cities in China.

**Allergens**	**Overall P**	**Chengdu V Chongqing**	**Chengdu V Liuzhou**	**Chongqing V Liuzhou**	**Chengdu V Shenzhen**	**Chongqing V Shenzhen**	**Liuzhou V Shenzhen**
egg	0.00E+00	0.000099	6.05E-260	2.35E-236	6.48E-121	1.37E-95	8.04E-30
peanut	1.54E-62	7.74E-14	4.97E-17	0.458	5.68E-63	8.45E-24	1.13E-22
soybean	1.99E-207	6.26E-48	4.99E-190	1.77E-64	7.98E-75	0.000002	3.38E-24
cow's milk	0.00E+00	0.036	4.63E-299	8.92E-297	6.11E-155	3.14E-146	6.56E-31
beef	0.00E+00	1.27E-09	7.98E-187	6.91E-161	1.10E-176	3.16E-145	0.517
mutton	0.00E+00	0.7	1.51E-57	7.94E-59	1.60E-187	9.71E-194	1.04E-71
crab	3.54E-137	0.574	3.11E-74	9.11E-81	0.19	0.413	3.91E-65
shrimp	5.75E-66	0.002604	6.84E-14	0.000006	4.68E-56	5.57E-40	6.86E-22
fish mix	7.56E-35	0.771	5.78E-21	3.04E-20	0.116	0.065	1.39E-22
at least one food allergen	0.00E+00	8.14E-10	0.00E+00	0.00E+00	1.39E-304	9.84E-227	0.078
cat hair	7.30E-112	1.09E-30	1.57E-51	0.000096	3.06E-114	3.72E-37	3.88E-22
dog dander	7.45E-150	2.33E-59	3.81E-09	9.02E-34	1.62E-113	7.61E-15	9.46E-84
cockroach	0.00E+00	3.76E-76	0.00E+00	5.06E-196	6.40E-55	0.074	4.25E-159
*H*. japonicus	2.52E-275	0.065	1.41E-32	2.39E-26	3.82E-156	1.46E-149	1.61E-77
*A*. artemisifolia	8.52E-144	5.51E-12	2.04E-65	1.81E-29	3.72E-118	9.80E-71	1.93E-15
*A*. vulgaris	3.55E-89	0.105	2.72E-44	1.09E-36	2.10E-57	4.44E-49	0.000763
tree mix	2.27E-158	4.23E-14	3.20E-75	8.86E-34	9.06E-130	3.96E-76	4.41E-15
mite mix	0.00E+00	0.045	0.00E+00	0.00E+00	3.21E-83	3.96E-98	0.00E+00
house dust	3.60E-120	7.78E-102	4.88E-103	0.751	2.91E-29	2.01E-18	3.22E-18
mold mix	6.02E-131	0.000001	4.30E-35	1.35E-15	1.91E-103	7.95E-72	5.68E-30
at least one aeroallergen	0.00E+00	1.04E-57	0.00E+00	0.00E+00	1.02E-280	7.89E-103	2.04E-98

p<0.0083 (0.05/6) was considered statically significant.

We used Bonferroni method to correct the significant levels in a pairwise comparison of multiple samples.

The positive rates of cat hair, *A. artemisifolia*, tree mix, and mold mix were the highest in Shenzhen, followed by Liuzhou, Chongqing, and Chengdu (*p* < 0.0083). The positive rate of mite mix was the highest in Liuzhou, the lowest in Shenzhen, and similar in Chongqing and Chengdu (*p* > 0.0083). The positive rate of house dust was the highest in Liuzhou and Chongqing with no difference between them (*p* > 0.0083), followed by Shenzhen and Chengdu (Figure 6B and Table 8).

## DISCUSSION

We found the top five allergens in our study were mite mix, cockroach, crab, fish mix and house dust, these findings agree with an epidemiological survey in southern China that found house dust mite (28.1%) and cockroach (24.3%) to be the most important inhalant allergens, while the most important food allergen was crab (15.8%) [12]. We found a low rate of allergy to cat hair and dog dander in our cohort, possibly due to the low pet ownership rate in our country. A recent multicenter study in north China reported that the highest positive rates were of *A. vulgaris* and *A. artemisifolia* [13]. Allergy to peanuts and eggs seems to be common in Northern Europe, the United States, Canada, and Australia [14]. These discrepancies could be due to differences in geographic location, climate, living environment, ethnicity and eating habits.

Almost all the general sensitization trends decreased with age, especially from infants to middle age. This could be explained by the immature development of the digestive and respiratory systems and the more active immune system at a young age. When children grow older, their gastrointestinal and respiratory tracts develop, eventually leading to lower allergy rates. Recent studies revealed that intestinal microbiology is not yet established in children aged 0–5 years and that the intestinal bacteria could affect the immune function of the gut and, in turn, affect the allergy to food [15, 16]. The digestive enzymes of infants ingesting food allergens are not well-formed or secreted, resulting in allergic symptoms. Similarly, the respiratory tract mucosa in infants is not well developed, possibly leading to the high positivity rate to aeroallergens. This phenomenon prompted people to read the food ingredient list carefully when feeding babies [17]. The recent Enquiring About Tolerance (EAT) study showed that an early food intervention was effective for allergic diseases [18]. However, the positive rate of the single allergens crabs and cockroaches was much lower in the 0–10 age group than in the 11–20 one. This difference might be due to the differences in ingested volume and contact frequency, as parents in China tend to control what and how much their children eat or contact. A smaller ingestion volume and lower contact frequency might lead to a lower allergic impact on children. Moreover, the rate of allergy to aeroallergens was always higher than to food allergens in the 0–10, 11–20, 21–30, and 31–40 age groups. We suggest that the widespread aeroallergen distribution is unavoidable, unlike food allergens.

Many inhaled or food allergens in this study showed significant sex differences, with males consistently showing higher positive rates (OR > 1, *p* < 0.0025). However, several studies have suggested that male children and adults have higher total and allergen-specific IgE levels than female [19, 20]. Although not all studies on IgE levels in children have observed male predominance [21, 22], the data suggest that very young males are more prone to develop IgE sensitization following allergen exposure than females [23]. The mechanism for these differences is not clear but could be attributed to genetic differences or differential sex hormone exposure in utero or postnatally [24]. Clinical studies showed a sexual dimorphism in asthma along different hormonal points of life, Shah R et al. [11] found that before puberty, asthma symptoms were higher in boys than in girls. Interestingly, although males still displayed higher IgE levels than females after puberty [25], females were either at an equal or higher risk of developing allergic diseases [26–28]. Indeed, a UK study that surveyed the presentations to medical clinics after puberty reported that females presented more often with asthma-, rhinitis-, and eczema-associated symptoms than males [29]. Together, these results suggest that IgE sensitization should be distinguished from clinical allergic diseases.

We found that the detectable rate of allergen-specific IgE differed between seasons, possibly due to the allergen exposure rate. The allergen-specific IgE for cat hair and dog dander was the highest in the spring, possibly because it is the depilation season. Mite mix, house dust had the highest positive rates in the summer due to the warm and wet climate in southern China. We attributed the surging numbers of allergies to crabs in the autumn to the Chinese mitten crab consumption during this season. The positive rate of *H. japonicus*-specific IgE in the autumn was higher than in the other seasons, which was consistent with the natural law of pollen propagation.

Because of their similar diet and living habits, we found no difference between Chengdu and Chongqing in the rate of IgE for single allergens such as cow’s milk, mutton, crab, fish mix, mite mix, *A. vulgaris*, and *H. japonicus* (*p* > 0.0083), and the top three allergens in both were mite mix, crab, and fish mix. However, the serum house dust allergen-specific IgE positive rate in Chongqing was higher than in Chengdu, possibly because Chongqing is close to the mountains.

The most common allergens in Chengdu, Chongqing, and Liuzhou were consistent with those in the entire study population (mite mix, cockroach, house dust, crab, and fish mix), but the positive rates to allergens such as egg, cow’s milk, and soybean in Liuzhou was consistently higher than the other cities (9.35–10.14%). We suggest that the cause of this is the fact that the mean age (25.55 ± 19.28 years) of the participants in Liuzhou was the lowest of the four cities, and cow’s milk and egg white sensitization prevalence in children could be as high as 41–67% [30]. Comparing all 19 allergens between the four southern cities, the proportion of positive IgE to food allergens and aeroallergens in Liuzhou was the highest, especially for cockroach and mite mix (*p* < 0.0083), possibly due to the hot and wet climate there. The prevalence of sensitization to mites was higher in subtropical and tropical areas where house dust mites were abundant due to the higher humidity and ambient temperature in these environments [31]. Sensitization to fish mix in Liuzhou rose again in the 61–70 and 71–90 age groups (*p* < 0.05), which we attribute to the chaotic inner environment in older people [32].

The similar rate in all allergens in Shenzhen (5.33–6.18%), and the lowest of the four cities, was an astonishing result. This could be explained by the fact that Shenzhen is a coastal city in southeast China. Furthermore, Shenzhen has a dry climate throughout the year but in the summer. The temperature there remains around 22.4° C throughout the year, which is not conducive to the growth of mites. Besides, Shenzhen residents are accustomed to consuming aquatic products such as crabs, shrimps, and fish, so they are less sensitive to them.

However, being positive to allergen-specific IgE does not necessarily mean being allergic to it. The cross-reactive carbohydrate determinants (CCDs), which are widespread in nature, lead to antigenic cross-reactions that could cause false positives results in the allergen test. Yang et al. [33] showed that shrimp is a common allergic food in southern China, and the higher proportion of shrimp sensitization in rural subjects could be due to cross-reactivity to cockroaches. While this is possible, we did not exclude the interference of CCDs in our study.

In summary, we found that almost all allergens peaked in young participants, the positive rates of aeroallergens were always higher than of food allergens in the young and middle age groups, and several allergens (mite mix, tree mix, cockroach, crab, shrimp, and fish mix) showed significant sex differences, with males being at a higher risk. Moreover, the prevalence of allergen variety changed with seasons and between regions, possibly due to differences in lifestyle, climate, and geomorphological features. Our research reminds us to take various factors (age, sex, season, region, ethnicity and diet habit) into account when diagnosing allergic diseases and determining the allergen type that caused them or actively preventing the impact of these allergens on susceptible populations. The study results are significant for clinical practice and public health. Although further validation and interpretation are needed, the number of samples in our multicenter study was larger than other epidemiologic surveys in China. Our findings contribute to data for evidence-based management of local allergies in China and worldwide.

## MATERIALS AND METHODS

### Participants

We recruited 55,432 participants who underwent specific IgE tests between January 2007 and December 2019 from Chongqing (14,612, 26.36%), Chengdu (14,030, 25.31%), Liuzhou (17,217, 31.06%), and Shenzhen (9,573, 17.27%). These participants included 27,408 males (49.45%) and 28,024 females (50.55%); the mean participant age was 33.78 ± 19.10 (range, 2–90) years. The participants were divided into eight age groups to explore differences associated with age: 0–10 years (9,456, 17.06%), 11–20 years (5,450, 9.83%), 21–30 years (8,229, 14.85%), 31–40 years (10,011, 18.06%), 41–50 years (11,785, 21.26%), 51–60 years (6,058, 10.93%), 61–70 years (3,085, 5.57%), 71–90 years (1,358, 2.45%). The institutional review board and ethics committee of Sichuan University approved this study. All participants or their guardians provided written informed consent.

### Blood sampling

We collected blood samples (3 mL) from all participants into BD Vacutainer blood collection tubes containing sodium heparin. All blood samples were stored at 2–8° C for a maximum of 14 days pending testing. All samples were incubated at room temperature (18–25° C) for 30 min before testing.

### Allergen-specific IgE analysis

Allergen-specific IgE was tested using the EUROBlotMaster (EUROIMMUN, Luebeck, Germany) with the Euro11 Allerg EL60 and EUROLINE Atopy China (IgE) kit. We tested serum IgE specific to nine food allergens [egg, peanut, soybean, cow's milk, beef, mutton, crab, shrimp, fish mix (cod, scallop, salmon, and carp)] and ten aeroallergens [cat hair, dog dander, cockroach, *H. japonicus*, *A. artemisifolia, A. vulgaris*, tree mix (willow, poplar, and elm), mite mix (*Dermatophagoides farinae* and *Dermatophagoides pteronyssinus*), house dust, and mold mix (*Penicillium notatum, Cladosporium herbarum, Aspergillus fumigatus,* and *Alternaria alternate*)]. At least two skilled technicians in each city evaluated the test results by comparing the signal position on the incubated test strips with the printed evaluation strips. Positive bands indicated the presence of IgE for a specific antigen.

### Statistical analysis

Descriptive analysis, χ^2^ tests, and OR assessment was performed by IBM SPSS Statistics for Windows, Version 25.0 (IBM Corp., Armonk, NY, USA). The χ^2^ tests analyzed differences in the distributions of allergens between groups. Differences with *p* < 0.05 were considered statistically significant. However, differences between male and female participants were considered statistically significant at *p* < 0.0025 (the *p*-value was reduced due to the large sample size after dividing the participants into the two sex groups). We used the Bonferroni method to correct the significance levels for multiple comparisons (*p* = 0.05/n). All figures were generated using GraphPad, Version 6.0(GraphPad Software Inc., San Diego, CA, USA.).

### Data availability

The data and materials analyzed in this study are available from the corresponding author upon reasonable request.

## Supplementary Material

Supplementary Figures
