# Supplementary material for: Prevalence of allergen-specific IgE in southern China: a multicenter research
Source: Aging (Albany NY). 2021 Jul 22;13(14):18894–911. doi: 10.18632/aging.203341 (PMC8351705; doi:10.18632/aging.203341)
Supplement: Supplementary Figures [file aging-13-203341-s001.pdf]

SUPPLEMENTARY FIGURES

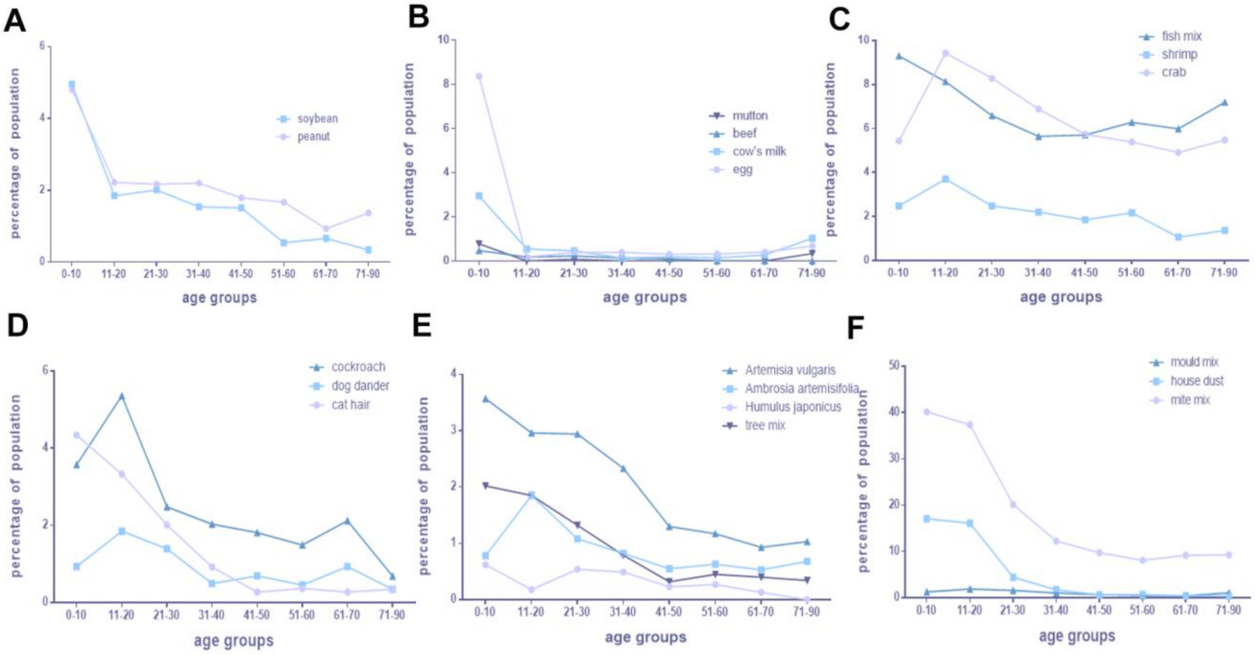

Supplementary Figure 1. The difference of the positive rate of various allergens in different age groups in Chengdu. (**A**–**C**) Food allergens. (**D**–**F**) Aeroallergens.

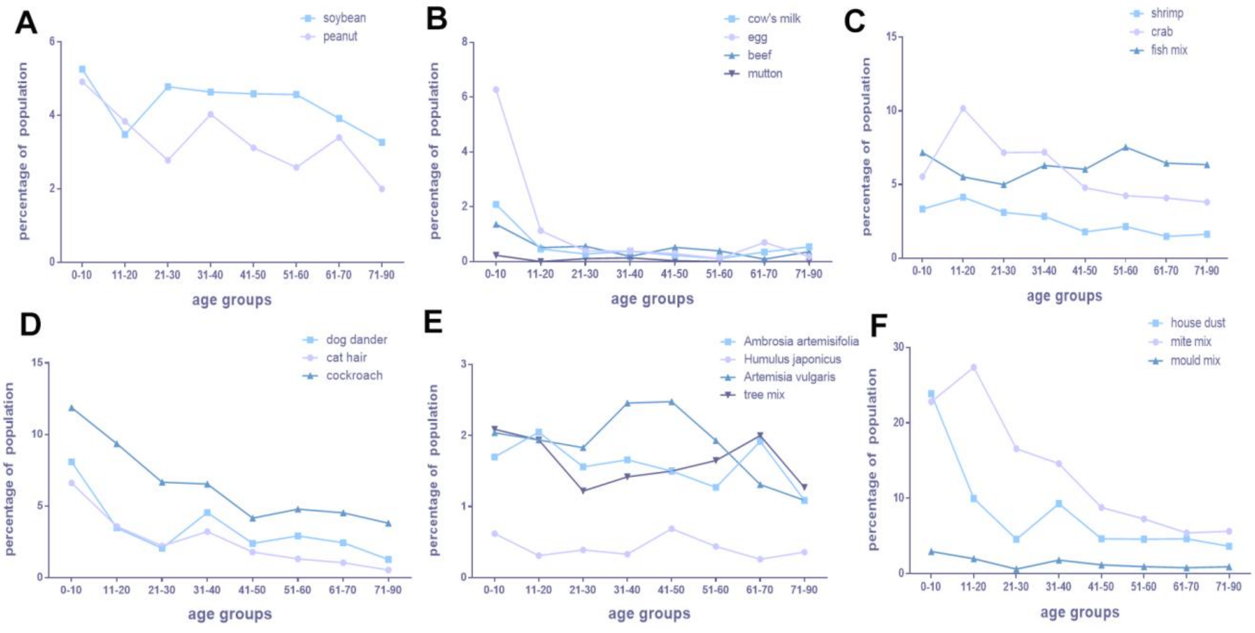

Supplementary Figure 2. The difference of the positive rate of various allergens in different age groups in Chongqing. (**A**–**C**) Food allergens. (**D**–**F**) Aeroallergens.

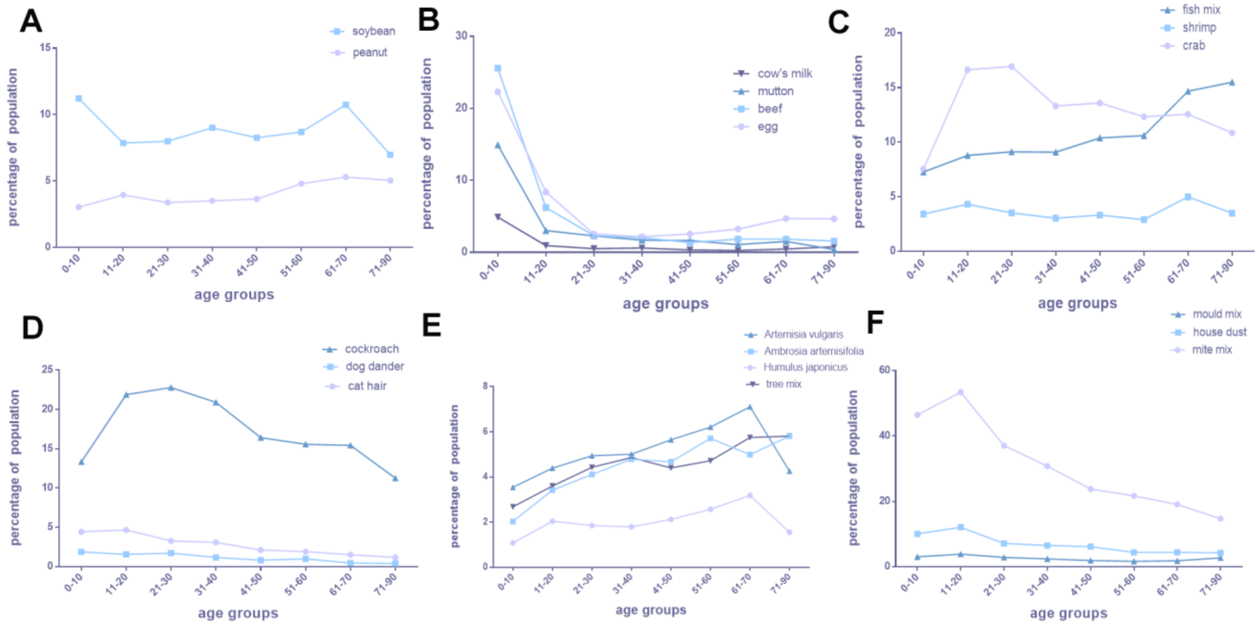

Supplementary Figure 3. The difference of the positive rate of various allergens in different age groups in Liuzhou. (**A**–**C**) Food allergens. (**D**–**F**) Aeroallergens.

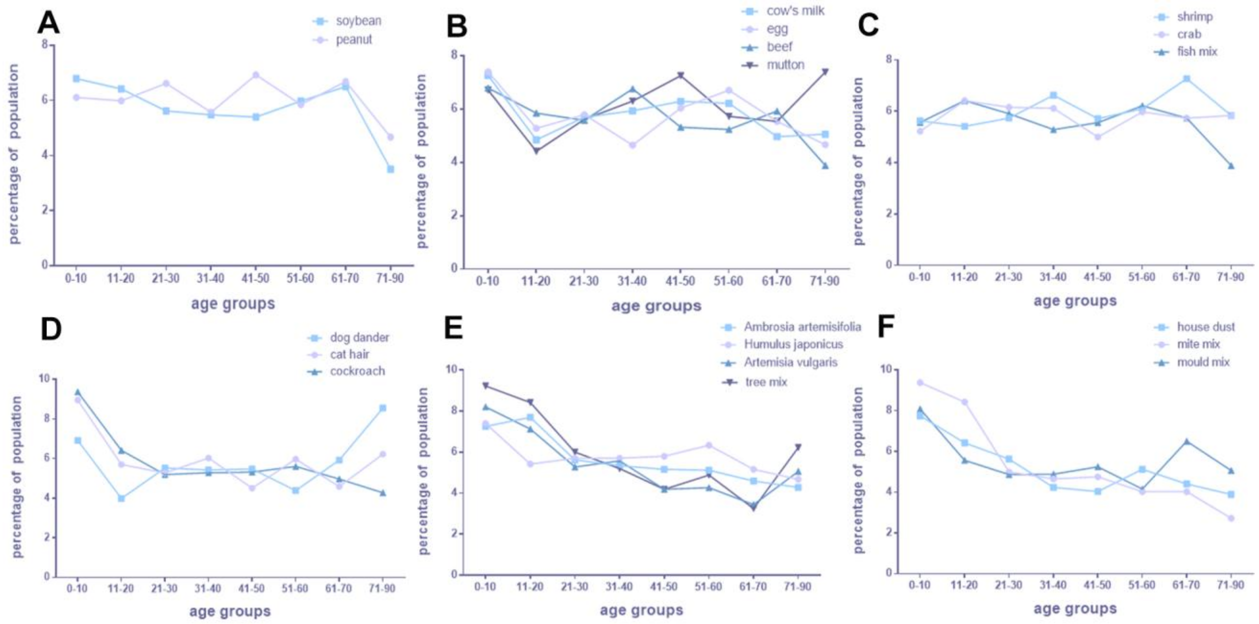

Supplementary Figure 4. The difference of the positive rate of various allergens in different age groups in Shenzhen. (**A**–**C**) Food allergens. (**D**–**F**) Aeroallergens.

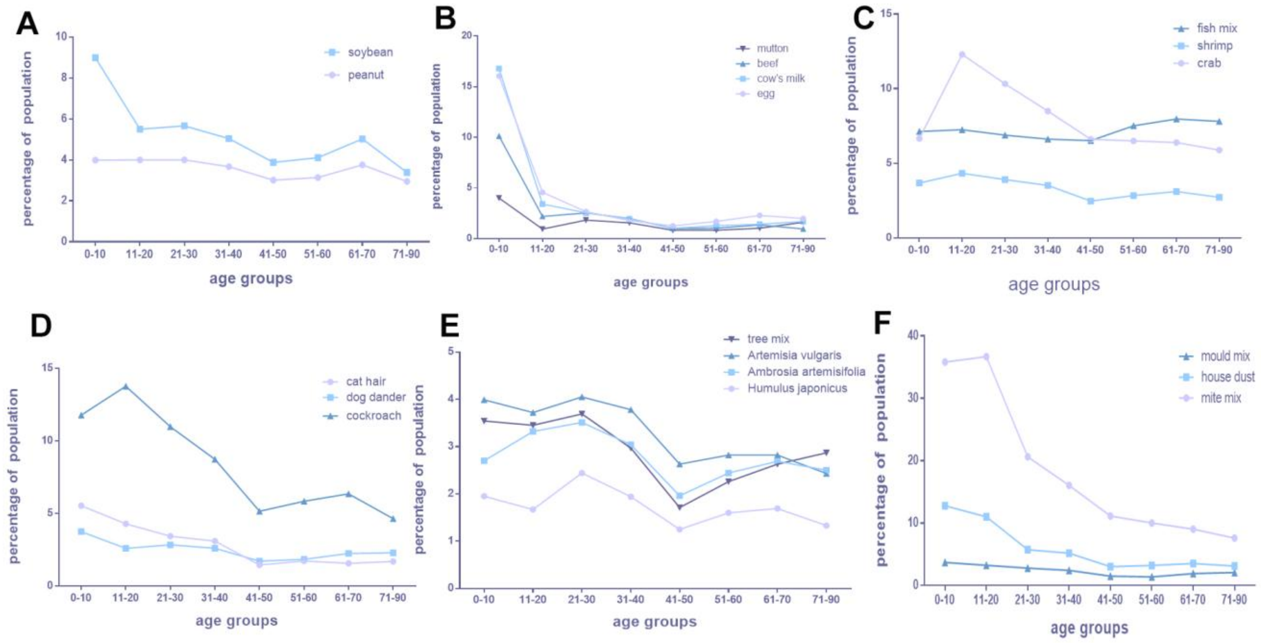

Supplementary Figure 5. The difference of the positive rate of various allergens in different age groups overall these four cities. (**A**–**C**) Food allergens. (**D**–**F**) Aeroallergens.
